# Modified Morita Therapy for Treating Hospitalized Patients with Depression

**DOI:** 10.62641/aep.v53i2.1714

**Published:** 2025-03-05

**Authors:** Haiqun Shen, Yiyu Mao, Yu’e Fei, Yuchao Lu

**Affiliations:** ^1^Department of Clinical Psychology, Kangci Hospital of Jiaxing, 314500 Tongxiang, Zhejiang, China; ^2^Department of Rehabilitation, Kangci Hospital of Jiaxing, 314500 Tongxiang, Zhejiang, China

**Keywords:** Morita therapy, depression, social functioning, quality of life

## Abstract

**Background:**

Depression is a common mental illness worldwide. Morita therapy is a novel and effective intervention method for treating depression patients. This study aimed to investigate the effects of modified Morita therapy on social functioning and quality of life in individuals suffering from depression.

**Methods:**

The data of depressive patients hospitalized in Kangci Hospital of Jiaxing from June 2021 to May 2022 were collected and analyzed by propensity score matching (PSM). The control group received antidepressant treatment and standard psychiatric care (n = 30), while the study group received modified Morita therapy on the basis of standard treatment (n = 30). Both experimental groups received a six-week intervention. The 17-item Hamilton Depression Scale (HAMD-17) was used to assess the severity of depression before and after the intervention. Hamilton Anxiety Scale (HAMA) was used to assess the anxiety level of patients. Social Dysfunction Screening Scale (SDSS) was used to evaluate the social functioning of the patients. Generic Quality of Life Inventory-74 (GQOLI-74) was implemented to evaluate the quality of life of patients based on four dimensions: physical function, psychological function, social function, and material life status through 74 items.

**Results:**

After six weeks of intervention, the study group exhibited significantly reduced HAMD-17, HAMA, and SDSS scores compared to the control group (*p* < 0.05). Furthermore, significant improvements were observed in physical function, psychological function, social function, material well-being domains, and the overall GQOLI-74 questionnaire scores within the study group compared to the control group (*p* < 0.05).

**Conclusion:**

Modified Morita therapy effectively alleviates depression and anxiety levels among depressed patients while enhancing their social functioning and improving their quality of life, thus highlighting its clinical applications.

## Introduction

Depression is a prevalent mental illness characterized by clinical symptoms such as low mood, anhedonia, fatigue, and reduced energy levels [1]. It has a protracted onset period, is challenging to treat, is prone to relapse, and is associated with high disability and suicide rates. Consequently, it has emerged as a significant public health concern in contemporary society [2]. According to the World Health Organization (WHO), there are approximately 350 million individuals worldwide who suffer from depression. By 2030, it is anticipated that depression will be the leading cause of disease burden globally [3]. The China Mental Health Survey indicates that the lifetime prevalence rate of depressive disorder among Chinese adults is 6.8%, with 3.4% suffering from depression, amounting to 95 million patients in China [4].

Currently, pharmacotherapy is the primary treatment for depression. However, its limitations include prolonged treatment duration and substantial adverse effects that may compromise patient compliance [5]. Psychotherapy, as a critical adjunctive therapeutic approach, aims to reduce medication side effects and the recurrence rates of depressive episodes, and has demonstrated considerable efficacy [6]. Nevertheless, existing psychological treatments are often cumbersome and costly, lacking convenient and cost-effective alternatives that enhance social functioning and quality of life for individuals with depression.

Morita therapy is a novel and effective intervention capable of stimulating patients’ existential awareness while improving their social functioning and overall quality of life through phased operational strategies [7]. Originally developed for treating various neuroses and schizophrenia disorders, this study employs a modified version of Morita therapy to treat depressed patients and observe its impact on their quality of life and social functioning.

## Materials and Methods

### Study Participants

This single-centre retrospective cohort study included 196 patients with confirmed depression who were hospitalized at Jiaxing Mercy Hospital between June 2021 and May 2022. The inclusion criteria for this study were as follows: (1) patient aged between 18 and 60 years, with no gender restrictions; (2) diagnosis of depression based on the International Classification of diseases-10 (ICD-10) [8] criteria; (3) no history of significant organ diseases or tumors affecting heart, liver, kidney or other vital organs; (4) individuals with education levels of junior high school or above; (5) no hearing, vision, and language communication disorders. However, patients with severe personality disorders and end-stage cardiovascular and cerebrovascular diseases were excluded. Informed consent forms were provided to all participants who voluntarily agreed to participate in this study after being informed about the study’s purpose and procedures. The hospital ethics committee (No. 2021-020) approved these consent forms. Furthermore, the study was performed in compliance with the Declaration of Helsinki.

### Propensity Score Matching (PSM)

The propensity score which is the conditional probability of being treated under the covariate condition, can reduce bias and equalize confounding factors between groups. The propensity score covariates in this study included age, gender, duration of disease and 17-item Hamilton Depression Scale (HAMD-17). The propensity score was calculated by logistic regression analysis using the R software MatchIt package (R Foundation) and 1:1 nearest-neighbor matching without a caliper value. After matching, P values for the group samples were all greater than 0.05, indicating a good balance.

### Treatment Procedure

The control group was treated with conventional drugs, specifically Venlafaxine hydrochloride sustained-release capsule (National Drug Approval Number: J20120038, Wyeth Pharmaceutical Co., Ltd., Suzhou, China) at a dosage of 75 mg once daily in the morning. After one week of continuous treatment, the dosage was adjusted based on the patient’s condition, generally increased to 150–225 mg once daily. Each course of treatment lasted four weeks, with two continuous courses. However, the study group received modified Morita therapy for six weeks.

The implementation of improved Morita therapy consisted of four stages [9]:

(1) Relative bed rest period: Patients were directed to stay in bed for at least 12 hours a day and attended Morita lectures twice a week for 7 days. The stage aimed to provide physical and mental relief, enabling patients to confront distressing situations and reality with an enhanced understanding of Morita theory and its treatment principles.

(2) Light operation period: Patients were encouraged to sleep for 7–8 hours at night and engage in voluntary activities during the day. This stage primarily involved homework therapy with low-intensity indoor activities such as music association, reading, puzzles, picture music, self-analysis, sand painting, art appreciation, flower arranging, origami, and imaginary painting. Additionally, patients were encouraged to maintain daily diaries for reflection throughout the 14-day course. This stage aimed to inspire patients’ enthusiasm towards life and redirect their focus back to real-life experiences.

(3) Heavy operation period: Building on the second phase, this stage involved increased physical activity through exercises like Jiamusi aerobics, five elements’ aerobics, physical games, table tennis, badminton, and fitness activities. These activities aimed to enhance patient autonomy and initiative by encouraging weekly record-keeping for treatment summaries over 14 days. Moreover, the goals included developing concentration skills, experiencing success, and enhancing self-confidence.

(4) Social adjustment training period. This stage of treatment focused on daily life and social adaptation training, including group sessions for life adaptation, emotional management, mental health education, motto therapy, role training, social skills training, and other crucial skill development. Moreover, it aimed to guide the formulation of future life and work plans while completing the treatment summary. This stage lasted 7 days, aiming to foster initiative and coordination.

### Evaluation Indices

The study participants underwent comprehensive assessments using the following evaluation tools before and after the intervention:

(1) The HAMD-17 [10] was utilized to assess the severity of depression among patients. A score >24 indicated severe depression, 17 < X ≤ 24 indicated depression, 7 < X ≤ 17 suggested suspected depression, and ≤7 represented normalcy.

(2) The Hamilton Anxiety Scale (HAMA) [11] was employed to evaluate the level of anxiety in patients. Each item was scored on a five-point scale ranging from 0 to 4 points, with no symptoms scoring zero, mild symptoms scoring one point, moderate symptoms scoring two points, severe symptoms scoring three points, and extremely severe symptoms scoring four points.

(3) The social functioning of patients was assessed using the Social Dysfunction Screening Scale (SDSS) [12], consisting of ten items scored on a three-level scale ranging from 0 to 2 points. During this assessment, zero points indicated no abnormality or slight impairment, one point showed true functional deficiency, and two points indicated severe functional deficiency.

(4) The Generic Quality of Life Inventory-74 (GQOLI-74) [13] was implemented to evaluate patients’ quality of life, assessing health-related aspects across four dimensions: physical function, psychological function, social function, and material life status. The inventory included seventy-four items, each scoring between one to five points, with higher scores indicating better quality of life.

### Statistical Analysis

The statistical analysis was performed using SPSS 20.0 software (IBM, Armonk, NY, USA). Measurement data were denoted as mean ± standard deviation ($\bar{x}$ ± s), while counting data were expressed as n (%). Inter-group comparisons were conducted using independent sample *t*-tests, while intra-group comparisons were performed using paired sample *t*-tests. However, counting data were tested using the Chi-square test. Statistical significance was defined as *p* < 0.05.

## Results

### Baseline Characteristics of Patients before and after PSM

**Fig. 1.**
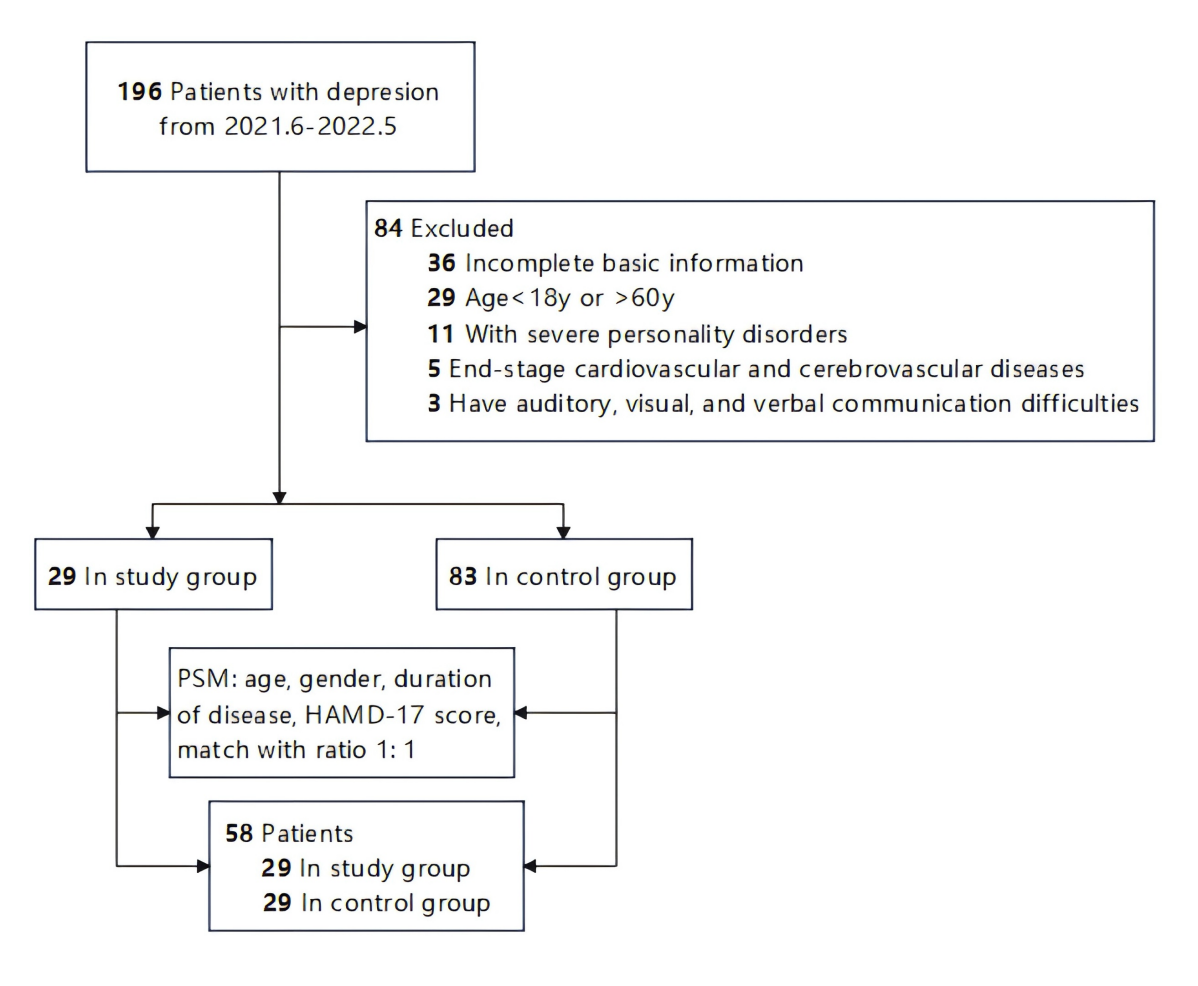
**Flow diagram of participant selection**. Note: PSM, propensity score matching; HAMD-17, 17-item Hamilton Depression Scale.

A total of 112 patients with depression were considered eligible for this study: 29 (25.89%) in the study group (receiving modified Morita therapy + primary therapy) and 83 (74.11%) in the control group. After PSM, 58 patients were enrolled in the study, 29 in the study group and 29 in the control group (Fig. 1). Table 1 summarizes the baseline characteristics of the selected study population before and after PSM. Before PSM, there were no significant differences in gender between the two groups, but there were significant differences in age, duration of disease and HAMD-17. After matching, there were no significant differences in age, gender, duration of disease and HAMD-17 score among all groups (*p* > 0.05), which was comparable.

**Table 1.** **Comparison of baseline characteristics between the two experimental groups ($\bar{x}$ ± s)**.

Item		Before PSM				After PSM			
		Control group (n = 83)	Study group (n = 29)	*t*/χ^2^	*p*	Control group (n = 29)	Study group (n = 29)	*t*/χ^2^	*p*
Age		46.13 ± 14.23	39.66 ± 12.00	2.190	0.031	36.90 ± 12.94	39.66 ± 12.00	–0.842	0.404
Gender				0.037	0.847			0.322	0.570
	Male	27 (32.53%)	10 (34.48%)			8 (27.59%)	10 (34.48%)		
	Female	56 (67.47%)	19 (65.52%)			21 (72.41%)	19 (65.52%)		
Duration		4.54 ± 1.68	2.72 ± 1.45	5.194	<0.001	2.54 ± 1.13	2.72 ± 1.45	–0.536	0.594
HAMD-17		31.04 ± 3.73	28.79 ± 4.16	2.713	0.008	28.38 ± 4.32	28.79 ± 4.16	–0.371	0.712

Note: HAMD-17, 17-item Hamilton Depression Scale.

One patient from each experimental group dropped out during the study (one due to incomplete data and one due to automatic discharge), resulting in 29 patients remaining in each group. In the study group, there were 10 males and 19 females, with ages ranging from 19–57 years (mean ± standard deviation: 39.66 ± 12.00 years). The disease course ranged from 0.5–5.8 years, with mean ± standard deviation of 2.72 ± 1.45 years. Moreover, the 17-item Hamilton Depression Scale score ranged from 21–36 points, with mean ± standard deviation of 28.79 ± 4.16 points. In the control group, there were 8 males and 21 females, with ages ranging from 18–58 years, with mean ± standard deviation of 36.90 ± 12.94 years. The course of disease ranged from 0.4–5.5 years, with mean ± standard deviation of 2.54 ± 1.13 years, and the HAMD-17 score ranged from 23–38 points, with mean ± standard deviation of 28.38 ± 4.32 points. There was no statistically significant difference in the baseline characteristics between the two experimental groups of depression patients (*p* > 0.05). Baseline characteristics of study participants are shown in Table 1.

### Comparison of HAMD-17 and HAMA Scale Scores between the Two Groups of Patients before and after Treatment

There was no significant difference in the HAMD-17 and HAMA scores between the two experimental groups before treatment (*p* > 0.05). After 6 weeks of treatment, the scores of HAMD-17 and HAMA in the two groups were significantly lower than those before treatment, and the scores in the study group were lower than those in the control group, with statistically significant differences (*p* < 0.05), as shown in Table 2.

**Table 2.** **Comparison of HAMD-17 and HAMA scores between the two groups of patients before and after treatment ($\bar{x}$ ± s)**.

Experimental groups	Case	HAMD-17		HAMA	
		Pre-treatment	Post-treatment	Pre-treatment	Post-treatment
Control group	29	28.38 ± 4.32	11.07 ± 2.67^*^	27.28 ± 3.43	12.93 ± 2.45^*^
Study group	29	28.79 ± 4.16	7.62 ± 2.56^*^	27.76 ± 3.73	8.48 ± 3.01^*^
*t*-value		–0.371	5.023	–0.513	6.177
*p*­-value		0.712	<0.001	0.610	<0.001

Note: ^*^ Compared to the group before the intervention, *p* < 0.001; HAMA, Hamilton Anxiety Scale.

### Comparison of SDSS Scores between the Two Groups of Patients before and after Treatment

Before treatment, there was no statistically significant difference in the SDSS scores between the two groups of patients with depression (*p* > 0.05). After 6 weeks of treatment, the SDSS scores of the two groups of patients were significantly lower than those before treatment, and the scores of the study group were lower than those of the control group, with statistically significant differences (*p* < 0.05), as shown in Table 3.

**Table 3.** **Comparison of SDSS scores between the two groups of patients before and after treatment ($\bar{x}$ ± s)**.

Experimental groups	Cases	Pre-treatment	Post-treatment
Control group	29	11.24 ± 2.43	9.79 ± 2.01^*^
Study group	29	10.97 ± 2.38	8.69 ± 1.93^*^
*t*-value		0.437	2.135
*p*-value		0.664	0.037

Note: ^*^ Compared to the group before the intervention, *p* < 0.05; SDSS, Social Dysfunction Screening Scale.

### Comparison of GQOLI-74 Questionnaire Scores between the Two Groups of Patients before and after Treatment

Before treatment, there was no statistically significant difference in the GQOLI-74 questionnaire scores between the two groups of patients with depression (*p* > 0.05). After 6 weeks of treatment, the scores of physical function, psychological function, social function, material life and GQOLI-74 questionnaire of the two groups of patients with depression were significantly higher than those before treatment, and the scores of physical function, psychological function, social function, material life and GQOLI-74 questionnaire of the study group were higher than those of the control group, with statistically significant differences (*p* < 0.05), as shown in Table 4.

**Table 4.** **Comparison of GQOLI-74 questionnaire scores between the two groups of patients before and after treatment ($\bar{x}$ ± s)**.

Experimental groups	Cases	Somatic function		Mental function		Social function	
		Pre-treatment	Post-treatment	Pre-treatment	Post-treatment	Pre-treatment	Post-treatment
Control group	29	45.31 ± 4.94	55.10 ± 7.02^*^	52.00 ± 4.31	58.28 ± 5.30^*^	53.55 ± 5.16	57.93 ± 6.74^*^
Study group	29	45.72 ± 5.66	64.00 ± 5.12^*^	52.79 ± 4.66	65.41 ± 4.03^*^	51.35 ± 4.89	66.35 ± 3.20^*^
*t*-value		–0.296	–5.513	–0.673	–5.770	1.671	–6.073
*p*-value		0.768	<0.001	0.504	<0.001	0.100	<0.001
Experimental groups	Cases	Material life		Total points			
		Pre-treatment	Post-treatment	Pre-treatment	Post-treatment		
Control group	29	44.66 ± 5.17	55.41 ± 7.08^*^	36.10 ± 3.73	49.86 ± 4.06^*^		
Study group	29	44.90 ± 6.42	64.97 ± 4.56^*^	35.86 ± 4.01	56.34 ± 4.11^*^		
*t*-value		–0.158	–6.105	0.238	–6.045		
*p*-value		0.875	<0.001	0.813	<0.001		

Note: ^*^ Compared to the group before the intervention, *p* < 0.001; GQOLI-74, Generic Quality of Life Inventory-74.

## Discussion

Modified Morita therapy is an individualized psychotherapeutic approach developed by Professor Masa Morita within Oriental culture [14]. It entails designing tailored training programs based on patients’ characteristics, utilizing action work to rectify personality traits, and ultimately enhancing psychological and social functioning. This therapeutic modality offers the advantages of simplicity, comprehensibility, convenience, and practicality [15, 16]. Building upon traditional Morita therapy, modified Morita therapy emphasizes on behavioral therapy and cognitive training for patients. Research has demonstrated that modified Morita therapy effectively ameliorates anxiety and depression [17, 18] while significantly improving social functioning [19].

In this study, after implementing modified Morita therapy in depressed patients, both experimental groups exhibited significant reductions in HAMD and HAMA scale scores compared to pre-intervention levels. However, the study group displayed a more pronounced decline. These findings indicate that modified Morita therapy effectively alleviates anxiety and depression among individuals with depressive disorders. The primary objective of improved Morita therapy is to help individuals let go of the past, confront current symptoms courageously, embrace present life circumstances wholeheartedly, and strive towards enhancing the quality of life, thereby achieving self-satisfaction and disease improvement. This therapeutic approach effectively mitigates negative emotions among patients while fostering positive affective experiences. Consequently, it bolsters subjective agency, enhances social communication abilities, eases somatization symptoms, and ultimately serves as an efficacious treatment for depression [20].

With the advancement of society, the concept of rehabilitation has significantly evolved. For patients with depression, there is an increasing focus on reducing depressive symptoms, improving quality of life, and restoring social functioning [21]. Despite being a psychological disorder, depression has an impact and causes harm to individuals than physical illnesses. Patients with depression often experience cognitive slowness and difficulties in interpersonal communication, leading to a decline in their social capabilities. These challenges are magnified in professional settings, where work efficiency is often compromised. Consequently, patients may become isolated from normal work groups and disconnected from society, ultimately affecting their overall social functioning. Researchers argue that prioritizing the restoration of social function and improvement in quality of life for depressed patients is more significant than merely eliminating symptoms [22, 23]. Therefore, it is crucial to explore and devote more attention to strategies aimed at reinstating patients’ quality of life and social function.

The findings of our study revealed that after a 6-week intervention, the SDSS score was significantly alleviated in the study group compared to the control group. Additionally, the scores for physical function, psychological function, social function, and material life dimensions, as well as the overall scores on the GQOLI-74 questionnaire, were substantially higher in the study group than in the control group. These findings suggest a significant improvement in social function and quality of life among patients in the study group. The modified Morita therapy, based on classical Morita therapy principles, relaxed restrictions on patients and incorporated occupational therapies such as music, painting, and sports tailored to individual situations, along with cognitive therapy techniques. By combining real-world activities that require engagement despite the symptoms experienced by patients during light or heavy tasks, this approach facilitated a shift of attention from self-focus to real-life experiences, redirecting mental energy towards external factors. Consequently, it enhanced patients’ ability to adopt positive thinking strategies for coping with negative psychological aspects associated with their illness. Implementation of Morita therapy allowed patients to gain deeper understanding and experience of concepts like “go with the flow and do what should be done” and “action changes personality”, thereby promoting improvements in maladaptive cognitive patterns and psychological coping abilities, which ultimately influenced their quality of life and social functioning.

## Conclusion

In conclusion, modified Morita therapy significantly ameliorates depression and anxiety levels while enhancing social functioning and overall quality of life. These findings underscore the clinical relevance and applicability of this therapeutic approach.

## Availability of Data and Materials

The data analyzed was available on the request for the corresponding author.
